# Efficacy and Safety of Photobiomodulation in MELAS: Protocol for a Series of N-of-1 Trials †

**DOI:** 10.3390/jcm14062047

**Published:** 2025-03-17

**Authors:** E-Liisa Laakso, Tatjana Ewais, Katie McMahon, Josephine Forbes, Liza Phillips

**Affiliations:** 1 Mater Research Institute, The University of Queensland, Aubigny Place, Raymond Terrace, South Brisbane, QLD 4101, Australia; josephine.forbes@mater.uq.edu.au (J.F.); liza.phillips@mater.org.au (L.P.); 2 School of Health Sciences and Social Work, Griffith University, Brisbane, QLD 4111, Australia; 3 School of Medicine and Dentistry, Gold Coast Campus, Griffith University, Southport, QLD 4215, Australia; tatjana.ewais@mater.org.au; 4 Mater Hospital Brisbane, Mater Misericordiae Limited, South Brisbane, QLD 4101, Australia; 5 School of Clinical Sciences, Queensland University of Technology, Brisbane, QLD 4000, Australia; k21.mcmahon@qut.edu.au

**Keywords:** mitochondrial disease, mitochondrial encephalomyopathy, lactic acidosis, stroke-like episodes, fatigue

## Abstract

**Background**: There is no cure for mitochondrial diseases which manifest in numerous ways including fatigue, muscle weakness, and exercise intolerance. Medical treatment varies and focuses on managing symptoms. Photobiomodulation (PBM) can decrease mitochondrial damage thereby increasing energy production and decreasing cell death. This pilot study will apply PBM to people with mitochondrial encephalomyopathy, lactic acidosis, and stroke-like episodes (MELAS) to examine the safety of application, and if changes occur in symptoms and signs after cross-over application/withdrawal of a sham or active PBM treatment including a two-week period of washout. **Methods**: This study is an exploratory, prospective series N-of-1 (single patient) studies. The protocol is guided by the CONSORT extension for reporting N-of-1 trials (CENT 2015), chosen due to the rarity of mitochondrial diseases, the fluctuating symptomology, and heterogeneity of the clinical presentation. The primary outcome is patient-reported fatigue assessed using the Checklist of Individual Strength and with concomitant evaluation of safety. Secondary measures are of depression, anxiety and stress, sleepiness, physical activity, blood lactate and creatine kinase, physical measures of sit-to-stand, and heel raise capability. Mitochondrial function will be evaluated using hydrogen magnetic resonance spectroscopy for lactate. PBM will be a participant-administered, home-based therapy using a multiple diode flexible array (BeniLight iLED-Pro Multi-Wave Multi-Pulse belt; 465 nm, 660 nm, 850 nm; average irradiance 5.23 mW/cm^2^; total joules: 770.1 J/treatment, all sites; 5 KHz; 20% duty ratio) over the anterior thigh muscles, posterior calf muscles and abdomen for 10 min to each site, three times/week. The safety of the intervention will be assessed. Descriptive statistics, causal analyses of time series data and dynamic modelling will be applied as relevant to the variables collected. Hydrogen magnetic resonance spectra will be acquired and averaged to obtain the content of the targeted hydrogen levels. **Discussion**: The study will provide guidance on whether and how to progress to a larger, randomised cohort study with sham control.

## 1. Introduction

Primary mitochondrial diseases (MD) are the most common group of inherited metabolic diseases and are characterized by impairment of the mitochondrial electron transport chain. The presentation of MD is complex, and diagnosis can be challenging due to the multi-system nature of these conditions. There is no curative treatment for MD, and management is supportive [1]. Although there are a number of mitochondrial and nuclear pathogenic variants associated with primary mitochondrial disease, the m.3243 A>G mutation in the *MT-TL1* gene encoding the mitochondrial tRNA^Leu(UUR)^ is one of the more common variants identified [2,3], and is associated with two primary, often overlapping syndromes:

1. Mitochondrial encephalomyopathy, lactic acidosis, and stroke-like episodes (MELAS) primarily affect the nervous system and muscles. MELAS presents in children or young adults as recurrent episodes of encephalopathy, myopathy with reduced exercise tolerance and fatigue, migraines, seizure disorder, and focal neurological deficits.
2. Maternally inherited diabetes and deafness (MIDD) is characterised by mitochondrial diabetes, hearing impairment, and maculopathy but can have other clinical manifestations and clinical overlap with MELAS.

This study focuses on MELAS which is progressive and life-limiting. Most individuals reportedly survive about 17 years [4] following the onset of symptoms such as seizures or other problems of the nervous system. There is an urgent need for the exploration of new treatment modalities to manage symptoms and improve the quality of life in people affected by MELAS. We aim to explore the notion that photobiomodulation (PBM; light therapy) activates mitochondria and relieves symptoms and signs of mitochondrial disease-related fatigue.

## 2. Background

MELAS is a rare, neurodegenerative, multisystem mitochondrial disease [2]. The function and survival of most mammalian cells are critically dependent on mitochondria. Although classically known to be the “powerhouse” of the cells, mitochondria are critical for other functions, including regulation of immunity, calcium balance, stem cell regulation, biosynthesis of hormones, cell death and renewal (autophagy/apoptosis), and cell metabolism [5].

Mitochondrial DNA (mtDNA) is maternally inherited and comprises 37 genes, encoding 13 proteins essential for oxidative phosphorylation (22 transfer RNAs and two ribosomal RNAs). However, the majority of the approximately 1500 proteins required for mitochondrial function are encoded by nuclear genes and as such are subject to classical Mendelian inheritance [6]. The most common pathogenic variant, known as m.3243 A>G was found to be carried by one in 500 community-based Australians [2]. The prevalence of all forms of childhood-onset (<16 years of age) MD has been estimated to range from 5 to 15 cases per 100,000 individuals [2].

A notable feature of mtDNA is heteroplasmy, the coexistence of multiple mtDNA variants within a single cell. The proportion of mutated to wild-type mtDNA can vary across different tissues, influencing disease manifestation [5]. The manifestations of mitochondrial disease are contingent on both the heteroplasmic load as well as the energy requirements of the tissue/organ systems involved, known as the “threshold effect” [7]. Due to mitotic segregation, the mutation load can change between different generations of cells and thus the threshold effect may vary over time resulting in variability in presenting features [6]. Symptoms of MELAS are most prominent in tissues with the highest energy requirements, including muscle [5]. Patients may present with fatigue, exercise intolerance, myalgia/cramping as well as an increased risk of rhabdomyolysis [8]. These muscle-related factors may severely impact quality of life, often out of proportion with clinical findings.

As there is no cure for MELAS, current standard treatment focuses on supportive management, including therapies for existing complications (e.g., cochlear implants, treatment of diabetes, cardiac failure, arrhythmia, migraines, or seizures), guidance on medications to avoid (e.g., sodium valproate, metformin, aminoglycoside, and other ototoxic medications), genetic counselling, family planning and cascade testing as well as instituting surveillance for timely management of potential complications [1]. There is minimal evidence for the use of a mitochondrial cocktail, including antioxidant therapy such as coenzyme Q 10 [1], but these therapies are sometimes trialled. The current clinical guidelines on physical exercise in mitochondrial disease [1] include recommendations for graded regular physical activity; for those without medical contraindications, moderate-intensity aerobic exercise at 70% of the person’s maximum heart rate to improve baseline levels of exercise; tailored exercise programs following consultation with exercise physiologist/physiotherapist/sports medicine therapist. Evidence to support resistance/concentric and high-intensity interval training in MD is limited [1]. We seek to add to the toolkit of therapies to address MELAS-related fatigue.

Although recently proposed by other authors as a therapy for MD [9], at the time of writing no published studies have investigated PBM as an effective method for managing symptoms of MELAS. We provide an overview herein, to understand why we think that PBM may be a useful adjunct in the clinical management of people with MELAS.

### The Proposed Role of PBM in MELAS

Mitochondrial disorders are primarily caused by dysfunctions in the oxidative phosphorylation (OXPHOS) system, which is crucial for ATP production in cells [8]. OXPHOS is a cellular process that generates energy in the form of adenosine triphosphate (ATP). Gorman and colleagues have described OXPHOS as a vital metabolic process occurring in the mitochondria of cells [10] and summarise the process as follows. OXPHOS requires the transport of electrons to molecular oxygen by the mitochondrial respiratory chain (known as the electron transport chain) involving four multi-subunit complexes (complex I to complex IV) and two mobile electron carriers known as coenzyme Q10 (or coenzyme Q or CoQ) and cytochrome *c*. The respiratory chain generates a transmembrane proton gradient harnessed by complex V (known as ATP synthase) to synthesize ATP. The electron transport chain is a substantial source of often deleterious reactive oxygen species (ROS). Although several enzymes, including superoxide dismutase (SOD) in mitochondria, are involved in ROS disposal, excessive ROS might have a role in the pathogenesis of MD [10]. The m.3243 A > G gene mutation causes translational defects that disrupt the OXPHOS function of the electron transport chain [11]. People with MELAS often exhibit a marked decrease in complex I activity, as evidenced by reduced enzymatic activity in muscle biopsies and fibroblasts [12].

There are a number of proposed mechanisms of action for PBM in the setting of mitochondrial dysfunction [13,14] as summarised here:

PBM acts via mitochondria displacing nitric oxide (NO) from the respiratory chain and increasing levels of adenosine triphosphate (ATP) with modest increases in superoxide dismutase (SOD) activity considered to be beneficial. PBM can produce ROS in normal cells but when used in oxidatively stressed cells, ROS levels and oxidative stress are lowered. The described changes activate transcription factors resulting in changes in gene expression and subsequent downstream production of chemical messengers implicated in the cellular improvements seen following PBM exposure clinically.

In vitro studies of the effects of PBM on mitochondrial activity have utilised various wavelengths (visible and invisible light), irradiances (power output per unit area of treatment), and fluences (photonic energy). Indeed, PBM has been found to increase the activity of respiratory chain complexes in an apparent dose- and time-dependent manner [15]. As defects in any one complex can influence other complexes [8], we propose that PBM may beneficially influence damaged mitochondria in people with MELAS. The best effects of PBM are likely to be in body tissues with increased numbers of mitochondria (such as muscles) using a combination of red, blue, and infrared wavelengths (e.g., [16]).

As pre-conditioning prior to exercise, PBM has moderately strong evidence for increasing the time to exhaustion [17] and low to moderately strong evidence for reducing signs of muscle fatigue in healthy subjects [18] but also in people with chronic obstructive pulmonary disease [19]. PBM significantly reduced muscle fatigue across a range of indicators including reduced ratings of perceived exertion (beneficial effect); increased electromyographic fatigue index (beneficial effect); and increased a range of muscle indicators including peak torque, time to peak torque, total work, average power, and average peak torque (beneficial effects), with significant differences between both PBM and placebo conditions [20]. In that study, when compared to a placebo, PBM applied before exercise improved indicators of muscle performance. A change in absolute deoxyhaemoglobin and myoglobin (deoxy[Hb + Mb]) after a PBM exercise pre-conditioning protocol was suggestive of muscle oxygenation and metabolism changes potentially by increasing local matching of bulk and microvascular oxygen delivery relative to muscle oxygen utilisation [21]. These effects may have been mediated by mitochondrial activity, but the study was not devised to test that effect.

Taken together, the literature suggests that muscle fatigue and poor performance are mitigated using PBM. We seek to test whether the PBM effects summarised above might be possible in MELAS, which is expressed clinically as muscle weakness and fatigue as a result of mitochondrial damage. The study rationale is that PBM stimulates mitochondrial biogenesis limiting the effects of muscle fatigue. We propose the mechanism of the PBM effect in MELAS is via enhancement of complex I activity [15] thereby improving fatigue, other measured symptoms/signs, and blood test results. We will undertake exploratory feasibility work to determine if mitochondrial function is a consequence of PBM when applied in people with MELAS. We start by testing for safety and any effects in a series of N-of-1 studies of people with MELAS under the supervision of the patients’ treating doctor (Author LP).

## 3. Objectives

Our primary objective is to determine if combined red (660 nm), infrared (850 nm), and blue (465 nm) wavelengths of PBM applied using an array of multiple light emitting diodes (LED belt) is safe and reduces patient-reported fatigue as measured using the Checklist of Individual Strength (CIS) in people with MELAS.

The secondary objectives are as follows:

‐ To determine if fatigue-related factors such as depression, anxiety, stress, and sleepiness are influenced by the application of PBM in people with MELAS.
‐ To determine if physical strength and activity changes in people with MELAS after PBM application to the large muscles of the lower limbs and the anterior abdomen.
‐ To determine if imaging using proton (^1^H) MR spectroscopy can show any change in mitochondrial activity in gastrocnemius muscles of the legs in people with MELAS.
‐ To determine if plasma creatine kinase (CK) and lactate are influenced by PBM application to the large muscles of the lower limbs and the anterior abdomen in people with MELAS.
‐ Based on outcomes, determine the feasibility of taking this research to the next phase as a larger clinical study.

## 4. Materials and Methods

### 4.1. Study Design

This study is an exploratory, prospective series of N-of-1 studies guided by the CONSORT extension for reporting N-of-1 trials (CENT 2015) [22]. We have chosen this method due to the rarity of MDs, their fluctuating symptomology, and the heterogeneity of clinical presentation. N-of-1 studies test the efficacy of an intervention using one or a small number of patients by sequentially applying and withdrawing the intervention [23]. The simplest of such study designs is an A-B-A design where A is ‘no treatment’ and B is the intervention being studied. Continuous measurement of chosen outcomes occurs through each of the stages.

**Figure 1:**
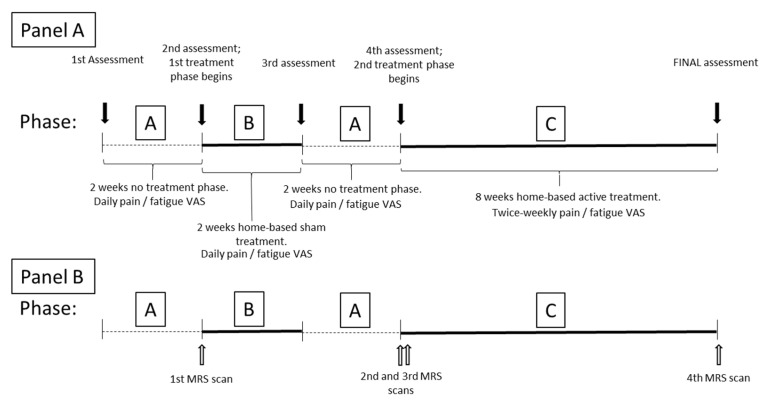
A priori design N-of-1 trial series (A-B-A-C phases). Panel A timeline: assessment time points and treatment phases. Panel B timeline: treatment phases and MRS scan time points. (A: no treatment observation phase; B: sham treatment phase; C: active treatment phase; VAS: visual analogue scale; MRS: magnetic resonance spectroscopy).

The PBM parameters will be standardised in all study participants, following an A-B-A-C design (Figure 1) as follows:

‐ A = no intervention observational period: Daily patient diary (visual analogue scale; VAS) of fatigue and pain to understand usual (baseline) behaviour of fatigue and pain symptoms, to compare with any change during/after intervention. Fourteen data points will be obtained in each non-intervention period. In this study, the repeated “A” period will also serve as a washout period after the sham phase, to account for any placebo response.
‐ B = sham intervention period: Daily patient diary (VAS) of fatigue and pain symptoms will be assessed. Fourteen data points will be obtained. We have included a sham phase in this series of N-of-1 trials to satisfy the requirement to understand if there may be a placebo element to the initial application of light. To reduce the burden on participants, we have chosen a 2-week sham period which is expected to demonstrate any change from baseline. The following A phase will assist in determining the durability of any placebo effect that we may need to consider in a future study.
‐ C = active intervention (PBM application) period: Twice-weekly patient diary of fatigue and pain symptoms will be assessed. Sixteen data points will be obtained over an 8-week period of treatment. The intervention period is based on exercise science findings demonstrating increased mitochondrial activity with endurance exercise [24] and mitochondrial biogenesis after 4–6 weeks of interval training in healthy individuals [24,25]. If PBM influences muscle mitochondrial function, we expect that 8 weeks of home-based intervention may be required to demonstrate effects in a clinical population that would normally struggle to participate in exercise programs.

As the latency, variability, and magnitude of the outcome of PBM in people with MELAS is not known, we have chosen to adjust the length of each phase based on the likelihood of the following: (a) gathering sufficient data points to observe an effect, and (b) garnering an effect with the study intervention.

### 4.2. Participants

Three patients from the Queensland Diabetes and Endocrine Centre (QDEC) who have been medically diagnosed with MELAS and where their primary symptom is fatigue. Participants will be over the age of 18 years and of either sex. Patients will be consented into the study non-concurrently. Patients will be ineligible to take part in the study if they have any contraindications or precautions to magnetic resonance spectroscopy or PBM (e.g., light sensitivity, pregnancy, recent history of cancer in area to be treated), uncontrolled psychiatric condition within the last 3 months, inability to comply with or undertake physical tests required for the study.

Consenting patients will receive a unique study code by which their data will be tracked. All study data will be de-identified at the conclusion of the study so that it becomes unidentifiable. Data collection tools will use both patient participant name and study code to permit tracking of data and extraction of relevant health information from medical records.

### 4.3. Intervention

We will assess the clinical response to the application of combined wavelengths of PBM using the BeniLight iLED-Pro Multi-Wave Multi-Pulse PBM device (https://benilight.com/products/benilight-iled-pro, accessed on 21 January 2025) which is an FDA-approved class 2 medical device. The light belt device is 385 mm × 85 mm in size. When programmed in active treatment mode, it emits 465 nm (visible blue), 660 nm (visible red), and 850 nm (infrared invisible) light via LEDs, and a custom lens package with >15° viewing angle; 5.23 mW/cm^2^ irradiance; 5 KHz frequency @20% duty ratio delivering a total fluence/treatment of 770.1 J. During the sham treatment period the LED array of the belt will be disconnected internally from the controller, so all functions display as required but with no output from the device.

The device will be self-applied by participants in the home setting, in contact with the skin over the anterior thigh muscles, posterior calf muscles and abdomen for 10 min at each site. The large quadriceps and gastrocnemius muscles have been chosen as these muscles are most likely to be affected by peripheral fatigue. The abdominal site has been chosen due to our recent (unpublished) findings that abdominally applied PBM has an effect on the gut–brain axis influencing factors associated with central (mental) fatigue.

Depending on the patient’s wishes, the scheduling of the treatment is modifiable, as follows:

‐ Six days/week with one day rest each week, alternating each application site (e.g., ten minutes to the abdomen on Monday and Thursday, ten minutes to each thigh on Tuesday and Friday, and ten minutes to each calf on Wednesday and Saturday)

OR

‐ Three days/week across all application sites (e.g., Monday, Wednesday, and Friday each week, the device is applied consecutively to the abdomen, the thighs, and calves for ten minutes at each site. The applications can be done in one sitting or spread across the day in separate ten-minute sittings).

The participant choice of application scheduling is permitted to fit in with the participants’ lifestyle but total PBM fluence and irradiance will be equivalent in both schedules.

### 4.4. Measures of Outcome

The primary outcome measure is patient-reported fatigue. The CIS is a 20-item self-report questionnaire that will be used to evaluate for changes in subjective experience of fatigue severity, and its three related domains of reduction in motivation, reduction in activity, and reduction in concentration (a de facto indicator for mental fatigue). The CIS has been used previously in studies of MD [26]. It has been shown to have good internal consistency (Cronbach’s alpha = 0.90 for total CIS, and between 0.83 and 0.92 for the individual scales) and moderate discriminant validity (CC 0.62) with high test–retest reliability (r = 0.74–0.86) [27,28].

To assess safety, participants will be asked to report/record in a study diary any unexpected effects (suspected side effects or adverse events) during the study. We will ask participants about such matters at each assessment point. Participants will have contact details of the investigators should they experience effects of concern.

Secondary measures will be assessed to aid in determining the feasibility of progressing the work into a group-based study because fatigue in mitochondrial disease is closely related to depression, anxiety, and sleepiness. These measures include the following:

1. DASS-21 to measure depression, anxiety, and stress (21 questions taking about three minutes to complete). All three scales of the DASS have been shown to have high internal consistency and meaningful discriminations in a variety of clinical and research settings. The short version, DASS-21, will be used in the study as it has shown validity comparable with the long version and will pose a lesser burden to complete for the participants [29].
2. The Epworth Sleepiness Scale (ESS) is a self-administered questionnaire with eight questions. Respondents are asked to rate, on a four-point scale (0–3), their potential for dozing off or falling asleep during eight different activities. The ESS score (the sum of eight item scores, 0–3) can range from 0 to 24. A higher ESS score indicates a greater inclination towards ‘daytime sleepiness’. The questionnaire takes no more than two or three minutes to answer and is available in many languages. The ESS has strong external criterion validity and a high level of internal consistency (Cronbach’s alpha = 0.88) [30].
3. To assess for any change in physical activity level, we will use the self-reported Habitual Activity Estimation Scale (HAES). The questionnaire is used to record time spent during a typical weekday and weekend, being “inactive” (lying down), “somewhat inactive” (sitting), “somewhat active” (standing or walking), and “very active” (sweating or breathing hard). The HAES is suitable for this study as it distinguishes between weight-bearing and non-weight-bearing activities, is feasible in clinical populations, sensitive to change, reliable and valid, has strong agreement with instrumented measures of physical activity, and high utility in a wide age range of free-living clinical populations [31].
4. Blood lactate and CK [32] will be analysed to determine if they are responsive to the intervention. Lactate is a major energy source for mitochondrial respiration thus an indirect indicator of the effect on mitochondrial activity. CK turns creatine into the high-energy molecule phosphocreatine, used by the body to generate energy. Any condition that interferes with muscle energy production or use increases levels of CK in the blood. These blood tests are standard and part of the routine assessment of people with mitochondrial diseases [1]. Blood tests will be required on three occasions, at intake to the study and at the start and end of active intervention Phase C.
5. We will test exercise tolerance on five occasions, using the following measures which focus on the leg muscles receiving the PBM treatment:
  ○ The 60 s sit-to-stand (60STS) test: This test is indicative of quadriceps muscle and gluteus muscle strength and endurance. Although it has not been used in mitochondrial disease, the 60STS test is simple and has been found to be sensitive in measuring the efficiency of rehabilitation in other diseases [33]. An improvement of at least three repetitions is consistent with physical benefits.
  ○ As the gastrocnemius muscle will be the focus of MR spectroscopy, we will assess calf muscle strength. There is no validated measure of calf muscle strength [34], thus we will use a simple measure of the number of heel raises that can be repeated by participants in 60 seconds.
  ○ If the participant uses a smart device (e.g., watch or ring), we will collect data on physical activity (step counts) across each phase of the study to assist in verifying participant activity levels as measured using the HAES.

Some participant medical history will be harvested from patient medical records to assist in confirming illness status for the purposes of potential publication of the N-of-1 trial series. As per CENT2015 [22], we will profile participant age, sex, confirmation of MELAS diagnosis, relevant co-morbidities, and age at MELAS diagnosis.

### 4.5. Magnetic Resonance Spectroscopy

We seek to understand if people with MELAS have impaired skeletal muscle metabolism (with reduced OXPHOS ATP production) which would affect physical performance and exercise capacity, thus impacting fatigue. To investigate this potential relationship, we will utilise hydrogen magnetic resonance spectroscopy (^1^H MRS) which is a non-invasive (surrogate) method to probe whether muscle affected by PBM alters mitochondrial function and is therefore more reliant on glycolysis. Lactate is a byproduct of glycolytic processes and can be detected by ^1^H MRS. Decreased lactate observed with ^1^H MR spectroscopy would support the notion that PBM increases mitochondrial activity.

MR spectroscopy will be carried out at the Herston Imaging Research Facility (HIRF) with a 3T PRISMA MRI (Siemens Healthineers, Erlangen). A ^1^H 18-channel flexible receive coil will be used for image and spectroscopy acquisition. Participants will be placed supine and feet first in a standardised position to avoid movement inside the magnet. T1 and T2 weighted anatomical images will be acquired axially from the mid-calf region to assist in the placement of the spectroscopy voxel. ^1^H MRS will be performed using a standardised single voxel position over the middle and distally towards the midline of the medial gastrocnemius calf muscle of the dominant leg. The medial gastrocnemius has been chosen because of the following: (a) it crosses 3 joints (knee, ankle, and subtalar joint) thus making it susceptible to fatigue during physical activity, (b) there is less variation in muscle fibre length in the medial gastrocnemius muscle compared to the lateral gastrocnemius muscle [35], and (c) the best separation of MR signals will be achieved in this bipennate muscle with its relatively uniform fibre orientation. To avoid the effects of chemical shift displacement, on-resonance acquisition for lactate will be used, and the reference signal will be measured separately. Ten resting ^1^H MR spectra will be acquired and averaged to obtain the content of the targeted hydrogen levels.

MR spectroscopy will be conducted four times as indicated in Figure 1. The MRS scan at time point 2 (after the initial 2-week observational Phase A) will be a baseline measure to establish the participant’s ‘usual’ level of target factors. A second and third MRS scan will be undertaken at the commencement of the active intervention Phase C. The second MRS scan will be taken immediately before the first active PBM treatment. The MRS will be followed by a 10-min PBM treatment of the calf muscles in a room adjacent to the MR scanner. The third MRS scan will be undertaken as soon as possible after the PBM treatment to determine if there is an ‘acute’/immediate change in mitochondrial activity signal following the treatment. After the completion of Phase C, the 8-week at-home self-applied PBM treatment, the final MR spectroscopy will be undertaken to determine if there is a longer-term change/response in mitochondrial activity signal and by how much (reflecting the rationale that light stimulates mitochondrial activity durability and biogenesis).

### 4.6. Research Ethics Considerations

The study has been ethically approved by the Mater Misericordiae Limited Human Research Ethics Committee (HREC/MML/108898). As this is an exploratory series of N-of-1 trials, there will be no randomisation of participants, and it is not considered essential in N-of-1 trials [22]. The order of intervention/withdrawal periods will not be randomised. Participants will be able to withdraw from the study at any time without a need for explanation. Where the participant requests withdrawal from the study, a request will be made for consent to use study data that has already been collected and permission to collect and use standard medical information for characterising the patient’s illness profile. No reference will be made in any publication or presentation that will allow the identification of a patient. The paper files and electronic files related to this study will be stored securely as per research compliance requirements. Once the study is completed, identifying data will be removed from all sources (redacted from paper-based forms and deleted from electronic forms) so that all data becomes unidentifiable. Imaging data will be stored using the patient’s unique identifying code on a secure cloud platform that is only accessible to the investigators.

### 4.7. Data Management/Analysis

As this is a preliminary series of N-of-1 trials, no power calculations have been performed. A valid sample size for the number of phases to be used in this study cannot be calculated with less than the planned three crossovers since the degrees of freedom for such a test are too small. There is no consensus on the most appropriate statistical method for different N-of-1 designs [36]. Descriptive statistics will be applied as relevant to the variables collected. Causal analyses of the time series data will be examined by graphical representation. Series meta-analysed results may be possible using dynamic modelling to account for unique data characteristics such as time trends and autocorrelation [36]. Due to the statistical challenges posed by N-of-1 trials, measures of pre-post effects may not be possible but these will be explored for the active intervention Phase C.

## 5. Discussion

This innovative study is expected to advance our understanding of whether PBM in MELAS is safe and if it influences subjective reports of fatigue and/or other markers of fatigue, related symptoms, and physical performance in people with MELAS. Additionally, we expect to garner information regarding the immediate and longer-term effects of PBM on muscle mitochondrial function in people with MELAS. The latter is an important focus as few studies have investigated and verified the effect of PBM on mitochondrial activity in real-time in humans.

The evidence for mitochondrial activation by light can be traced back more than 40 years to bench-top work by Passarella and colleagues [37]. By the 1990s and early 2000s, Karu and colleagues were measuring ATP output in in vitro studies implicating cytochrome C oxidase as the primary photoacceptor [38]. Others have provided further evidence of the role of light quanta in stimulating mitochondrial redox signalling and transduction [39,40]. Trajano and colleagues [41] provided an overview of mitochondrial dynamics in response to PBM in animal studies, but we have located only one report in humans which used MR spectroscopy to assess ATP changes after (transcranial) PBM [42].

The common genetic mutation of MELAS (3243 A > G) causes complex I deficiency causing muscle fatigue and is complicated by endothelial dysfunction with atherogenic and pro-inflammatory properties including upregulation of inflammatory genes, such as IL-6 and TNF-α [5]. Our choice of wavelengths (465 nm, 660 nm, and 850 nm) was made based on the work of authors who have investigated the in vitro and in vivo effects of different wavelengths of light on mitochondrial dynamics (e.g., [43,44,45,46]), muscle function (e.g., [47]), and inflammation [14]. For this study, 660 nm (red) has been chosen due to the findings of Osipov et al. [43] and Ferrarisi et al. [44] who found an increase in mitochondrial activity as indicated by increased oxygen consumption and mitochondrial membrane potential (MMP), respectively. The NIR wavelength was chosen based on findings that MMP kinetics increased [44,45], and myotubular ATP formed [44] after irradiation with wavelengths in the NIR band of the electromagnetic spectrum. NIR wavelengths have been found to have anti-inflammatory effects and to reduce oxidative stress [14], thus the combination of wavelengths is expected to have synergistic effects in managing the deleterious features of MELAS [48], including a reduction of oxidative stress.

The blue 465 nm wavelength has been chosen based on published results that blue light resulted in the highest effectiveness for stimulating oxygen consumption in the mitochondria [43]. Non-ultra-violet blue light has been shown to increase animal muscle growth by increasing satellite cell proliferation thus increasing the numbers of myocytes [49]. In in vitro studies, IL-6 and TNF-α decreased [50], verified by others who have shown that blue light down-regulates pro-inflammatory agents such as TNF-α, some interleukins and IFNγ, without inducing apoptosis [51].

Our study of PBM in people with mitochondrial disease is expected to add to the limited evidence regarding mitochondrial activity in humans before and after PBM. Our choice of timing of the post-intervention MRS scan was based primarily on the work of Silveira and colleagues [15] who found that activity in complex I in muscle mitochondria had increased five minutes after PBM. We are also interested in what the longer-term effects will be.

We will concentrate on lactate initially as it is over-expressed in MELAS and can be detected by ^1^H MRS. Should we find that PBM alters mitochondrial outputs using ^1^H MRS, we expect our future work may include ^31^P MRS to understand resonances more specifically from inorganic phosphate (Pi), phosphocreatine (PCr), adenosine triphosphate (ATP), and intracellular pH. Future work might also require measures of complex I deficiency as an outcome using skin or cheek fibroblasts or peripheral blood mononuclear cells.

We have chosen fatigue as our primary outcome measure based on our findings of PBM in young people with inflammatory bowel disease [52]. In an interim analysis, we calculated a large effect size for a reduction in participant-reported fatigue (using the FACIT-Fatigue scale) in response to abdominal and quadriceps muscle irradiation by laser PBM (at 904 nm).

### 5.1. Limitations

As MR spectroscopy is expensive, our initial work is being done as a series of N-of-1 studies to establish the proof of concept. As a series of N-of-1 trials, it will not be possible to entirely exclude a placebo effect, but we have attempted to account for the effect by incorporating an initial sham phase into the study design. Equally, the small number of participants means the findings cannot be generalised. It is important to note that there are many statistical challenges related to N-of-1 trials including variability in patient responses, treatment-by-patient interactions, and missing data related to non-compliance with treatment. By maintaining weekly contact with participants, we expect to reduce the incidence of non-compliance with treatment.

The rationale we have taken for the choice in PBM parameters is based on a mix of theoretical underpinnings and published effects. In the absence of previous literature, dosing characteristics (duration and frequency of application, and duration of treatment protocol) are theoretical. The choice of the nominated red, blue, and NIR wavelengths is based on results from published in vitro and animal studies. Whether any of these factors translate into a human model of MD is unknown.

### 5.2. Dissemination of Results

We expect to disseminate the results of this series of N-of-1 trials through publication in an appropriate journal and/or presentation at an appropriate international conference.

### 5.3. Implications for Practice

Our study has the potential to add support for using PBM as a feasible intervention for fatigue in MELAS and perhaps other chronic diseases expressing this debilitating and sometimes intractable symptom.

## 6. Conclusions

We expect the combination of participant-reported psychosocial, objective physical outcomes, and MR spectroscopy may provide insights into the relationship between central and peripheral fatigue and mitochondrial output and their responses to PBM. The results of this series of N-of-1 trials will aid in determining the value of progressing to further research, how to design such research, and calculating the sample size.

## Data Availability

Data will be available in de-identified form three months after the initial results publication, from the principal investigator, and subject to approval by the principal investigator.
